# BESST - Efficient scaffolding of large fragmented assemblies

**DOI:** 10.1186/1471-2105-15-281

**Published:** 2014-08-15

**Authors:** Kristoffer Sahlin, Francesco Vezzi, Björn Nystedt, Joakim Lundeberg, Lars Arvestad

**Affiliations:** Science for Life Laboratory, School of Computer Science and Communication, KTH Royal Institute of Technology, Solna, Sweden; Science for Life Laboratory, Department of Biochemistry and Biophysics, Stockholm University, Stockholm, Sweden; Science for Life Laboratory, School of Biotechnology, Division of Gene Technology, KTH Royal Institute of Technology, Stockholm, Sweden; Swedish e-Science Research Centre (SeRC), Department of Numerical Analysis and Computer Science, Stockholm University, Stockholm, Sweden

**Keywords:** Genome assembly, Scaffolding, Genome analysis, Mate pair next-generation sequencing

## Abstract

**Background:**

The use of short reads from High Throughput Sequencing (HTS) techniques is now commonplace in *de novo* assembly. Yet, obtaining contiguous assemblies from short reads is challenging, thus making scaffolding an important step in the assembly pipeline. Different algorithms have been proposed but many of them use the number of read pairs supporting a linking of two contigs as an indicator of reliability. This reasoning is intuitive, but fails to account for variation in link count due to contig features.

We have also noted that published scaffolders are only evaluated on small datasets using output from only one assembler. Two issues arise from this. Firstly, some of the available tools are not well suited for complex genomes. Secondly, these evaluations provide little support for inferring a software’s general performance.

**Results:**

We propose a new algorithm, implemented in a tool called BESST, which can scaffold genomes of all sizes and complexities and was used to scaffold the genome of *P. abies* (20 Gbp). We performed a comprehensive comparison of BESST against the most popular stand-alone scaffolders on a large variety of datasets. Our results confirm that some of the popular scaffolders are not practical to run on complex datasets. Furthermore, no single stand-alone scaffolder outperforms the others on all datasets. However, BESST fares favorably to the other tested scaffolders on GAGE datasets and, moreover, outperforms the other methods when library insert size distribution is wide.

**Conclusion:**

We conclude from our results that information sources other than the quantity of links, as is commonly used, can provide useful information about genome structure when scaffolding.

**Electronic supplementary material:**

The online version of this article (doi:10.1186/1471-2105-15-281) contains supplementary material, which is available to authorized users.

## Background

Recent high-throughput sequencing (HTS) technologies are attractive for *de novo* assembly projects since they produce millions of short DNA-sequences (referred to as *reads*) at low cost. However, these reads are only a couple of hundred base pairs long making it difficult for an assembler (*e.g.*, [1, 2]) to reconstruct the genome. As a result, the output of an assembly often consists of *contigs*, *i.e.*, subsets of reads assembled into longer fragments of genomic sequence.

**Figure 1:**
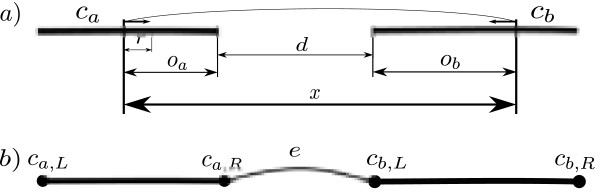
**Notation.** **a)** A read pair with insert size *x* (unknown distance) aligns to two contigs *c*_*a*_ and *c*_*b*_, thus creates a link between *c*_*a*_ and *c*_*b*_. The read pair gives rise to observations *o*_*a*_,*o*_*b*_ and they are used to infer the unknown distance *d*. Distances for *o*_*a*_,*o*_*b*_,*d* and *r* are illustrated. **b)** Graph structure and notations of the scaffold graph . Two contigs *c*_*a*_ and *c*_*b*_ connected by an edge *e* created from alignments of read pairs.

However, HTS-technologies provide protocols for creating read pairs that can be used to increase the contiguity of an assembly. We define a *read pair* as two reads that are sequenced at a known distance and orientation where the distance between the reads, is referred to as *insert size*. If the two reads within a read pair belong to different contigs *c*_*a*_ and *c*_*b*_, a *link* is created between *c*_*a*_ and *c*_*b*_, see Figure 1a. From this link, we can infer a relative order, orientation and distance between *c*_*a*_ and *c*_*b*_.

The process of linking and ordering contigs is called *scaffolding*. In addition to paired reads, information such as reference sequences of related organisms [3], restriction maps [4] and RNA-seq data [5], can be used for contig linking. However, reference based assembly is not applicable to most *de novo* sequencing projects, restriction maps are often not available, and RNA-seq data only have coverage over genes and contains no information about distance between reads which makes contig placement ambiguous. This makes read pair information the most commonly used (and often also the only applicable) source of information for scaffolding.

Unfortunately, scaffolding with read pairs poses challenges: reads may create spurious links because of read errors, heterozygosity and the repeated nature of the genome, and these spurious links make ordering and orientations among the contigs ambiguous. Hence, the scaffolding problem can be summarized as detecting and utilizing the correct links in order to find a consistent ordering and orientation of the contigs. The existing formalizations of scaffolding have been proven to be NP-complete, but it is still unclear if these formulations, even when finding the optimal solution with respect to the objective, solves the real (*i.e.* biological) problem. These approaches have focused on structural properties of the graph induced by contig links, with little emphasis on assessing correctness of individual links. Our approach focuses on removing incorrect links and employing sophisticated statistics to evaluate whether linking reads come from the underlying library distribution, or from misalignments. Only in a second step are structural properties used.

The following section discusses the formalization of scaffolding and related work, as well as gives an outline and motivation for our work. Our algorithm, realized in an implementation called BESST (Bias Estimating Stepwise Scaffolding Tool), is presented in detail in the Methods section. The algorithm scales well and is practical on very large and complex genomes, as proved by its use in the *Picea abies* genome project (20 Gbp) [6]. Furthermore, it excels at scaffolding with wider insert size distributions.

We present an evaluation of BESST against other popular stand-alone scaffolders on a large variety of datasets from GAGE [7]. Compared to previous assessments of novel scaffolding methodologies, the results obtained from our evaluation allows us to draw conclusions about the general performance of stand-alone scaffolders to a much higher extent. Another recent extensive evaluation of scaffolding tools is given in [8]. In our study we primarily compare stand-alone scaffolders because they have access to the same amount of information and are applicable in the same contexts (*e.g.* scaffolding with mate pair libraries that was not use in the original assembly). Nonetheless, we also include GAGE results on integrated scaffolders.

Our results indicate that no single scaffolder outperforms the others on all datasets although in total, BESST shows the most favorable results among stand-alone scaffolders. Furthermore, our algorithm outperforms other stand-alone scaffolders when the library insert-size distribution has a high standard deviation. Although there is wide performance variation around integrated scaffolders, overall, GAGE results demonstrate that Allpaths-LG’s assemblies scaffolded with its integrated scaffolder have the highest quality.

### The problem

#### Formalizing Scaffolding

As input for scaffolding we assume a set of contigs  produced by an assembler and a number of read pairs  from a read pair library that have been aligned to the contigs. These read pairs have an insert size distribution with mean *μ* and standard deviation *σ*. By aligning all reads in  on  we can define the graph  as follows: Each contig gives rise to precisely two vertices *c*_*i*,*L*_ and *c*_*i*,*R*_ in  where *c*_*i*,*L*_ denotes it’s 5’ end and *c*_*i*,*R*_ denotes it’s 3’ end (see Figure 1b). In a read pair, if  aligns to precisely one contig *c*_*k*_ and  aligns to precisely one contig *c*_*m*_, with *k* ≠ *m*, this read pair induces a relative orientation and an approximate distance between *c*_*k*_ and *c*_*m*_. This relationship is represented as an edge *e*, see Figure 1b. We let **V** and **E** denote the set of vertices and edges respectively in . Given , several formulations and methods have been proposed for scaffolding. We will discuss some of them below.

#### Problem formulations in related works

The scaffolding problem (SP) defined by *Huson et al.*[9] is a formulation that is commonly referred to. Using their notation, let  be defined as above and let *n* links between two contigs induce a weight *n* on the edge *e* between these two contigs. Furthermore, let *Φ* : *V* → *N* be an ordering, orientation and distance map of , that is, an assignment of non negative integer coordinates to the vertices *V* in  that preserves the contig lengths. Given such a mapping instance *ϕ*, [9] states that an edge *e* between *c*_*i*_ and *c*_*j*_ is consistent if *c*_*i*_ and *c*_*j*_ have the correct relative orientation (induced by aligned read pairs), and the distance between *c*_*i*_ and *c*_*j*_ is approximately correct. Here, approximately correct means that *e* suggests a distance between *c*_*i*_ and *c*_*j*_ that is less than *μ* + 3 *σ*, a heuristically chosen bound. If an edge does not satisfy these constraints, it is called inconsistent. Huson et al. [9] define SP to be the problem of finding a maximum weight consistent edge subset. SP has been used as foundation for a number of other works discussing scaffolding and proposed heuristics for solving it can generally be categorized as either “greedy” or “graph-structure” optimization algorithms.

Greedy algorithms proposed to solve SP include SSPACE and Bambus [10, 11]. SSPACE extends scaffolds in a greedy fashion applying a heuristic stopping criterion. Bambus builds scaffolds greedily with heuristics to remove inconsistent link constraints.

Graph-structure optimization algorithms that have been proposed to solve the SP are for instance: SOPRA [12] formulates a global optimization problem for solving relative contig orientation (exact for simple regions while a simulated annealing approach is employed in more complex regions of the graph). In a second step, read-pair distribution is used to determine the relative positions of contigs within a scaffold. If an inconsistency is found in the positioning step, the link causing the inconsistency is removed and the algorithm restarts at the orientation step. OPERA [13] builds scaffolds using the number of inconsistent edges *p* in a subgraph as a design criterion (the subgraph represents a potential scaffold). By treating *p* as fixed, they can obtain a polynomial time algorithm to find an optimal (with respect to a given *p*) solution to their slightly modified version of SP. The algorithm then tries all *p* starting from *p* = 0 and stops when a scaffold can be constructed. SLIQ [14] formulates a set of linear inequalities together with majority voting to predict placements of contigs. MIP Scaffolder [15] and GRASS [16] formulate SP as a mixed integer programming problem, but uses different techniques to find a solution. MIP Scaffolder resolves conflicting regions in the obtained MIP solution using heuristics such as removing edges that are stretched or contracted more than a given threshold. GRASS uses an Expectation-Maximization algorithm. The maximization step obtains degrees of penalties on contig links given fixed contig orientations. The penalties are set according to what magnitude the constraints for a link is violated. If a penalty is higher than a given threshold, the penalty of the link is “de-activated”, *i.e.,* its constraints are not considered. The expectation step is used to obtain the expected contig orientation of links given (the “activated”) penalties set in the maximization step. Links that are activated in the final solution are used for scaffolding.

There are advantages and disadvantages with these two classes of methods. Algorithms that are solving a local problem using a greedy approach often have better runtime and scale well on larger genomes but use oversimplified methods to find a solution which may only work for some genomes. Graph-structure optimization methods are instead hindered by their time complexity for finding a solution. The runtime scales poorly and it is difficult to predict if such an algorithm will ever finish on a larger dataset (see section Results).

Additionally, current methods that use insert sizes of paired reads for contig placement are built on false assumptions as we have previously shown [17]. This can complicate scaffolding when libraries with large insert-size variation are used.

#### Link inconsistency detection

The methods previously described define SP similar to [9] with modifications on how to define a consistent edge. Different heuristics are used between the methods to obtain a solution to SP. Yet, a common denominator is that the number of links supporting an edge is used as an indicator of reliability; edges with many links are preferred and those with few links are avoided. This reasoning is intuitive, but fails to account for variation in link count due to contig features. Firstly, the number of links between two contigs depends on the real (*i.e.*, biologically) distance between the two contigs and on their size [17]. Secondly, in SP we face structural features such as repeated regions, heterozygosity, and chimeric contigs. These features create clusters with reads being misaligned which cannot be seen as individual random events. It is our assumption that the number of random, non-structural, misalignments caused by, *e.g.*, sequencing errors are almost negligible compared to the structural misalignments. Link count is therefore a poor indicator of edge reliability.

We take a different approach to SP and, instead of link count, evaluate edges based on link statistics. When read pairs are mapped to contigs, are they placed on and connecting contigs in a reasonable way? In other words, we want to answer the question: given an edge *e*, is the cluster of read-pairs forming *e* coming from the read-pair library, or are they a consequence of a structural feature? If these reads together show similar properties as the read pair library we are scaffolding with (*e.g.*, mean, standard deviation), the edge is more likely to be correct.

We propose an algorithm, BESST (Bias-Estimating Stepwise Scaffolding Tool), that puts focus on analyzing the scaffold graph in local regions using statistics to filter out spurious edges created by structural errors. BESST starts scaffolding with contigs that meet a length criterion for the library (definition given in section Methods). It then continues with smaller contigs in an optional step. If several different paired-read libraries are used, BESST scaffolds with one library at a time in an increasing order of insert size of the library. Separating contigs with respect to size is mainly due to two reasons: **(i)** Links between large contigs make gap size estimation more stable (see [17]) giving a more robust statistical analysis. **(ii)** The gain in speed is significant since correct regions are simple path components in  which are found by visiting each edge once, thus, the time complexity is *O* (|**E**|).

## Results and discussion

*De novo* assembly validation is a task as difficult as *de novo* assembly itself. Recent evaluation efforts like GAGE [7] and Assemblathon [18] encountered several problems in identifying the best assembler. GAGE clearly demonstrated how the same assembler can have *completely* different performances (*e.g.*, quality) even on similar datasets (*e.g.*, bacterial genomes). This predicament was also supported in recent evaluation efforts [19, 20]. Despite this, as noted by [21], all new published assemblers and scaffolders have been compared to the then-existing tools highlighting better performances on a specific dataset using some specific metrics. We argue that evaluation of tools should be performed on multiple datasets and/or scenarios to avoid over-generalization and confirmation bias. For standalone scaffolders without stated dependencies, it is advisable to test on output from several assemblers to investigate overall performance.

We have tried to address the above issues in our evaluation of BESST, using a wide range of different datasets and assemblers. BESST has been compared with three other state-of-the-art scaffolders: OPERA, SOPRA, and SSPACE.

### Datasets

Evaluation has been performed using the three GAGE datasets [7] which gave us the possibility to evaluate scaffolders on three highly different genomes: *Staphylococcus aureus*, *Rhodobacter spaeroides*, and Human chromosome 14 (hereafter referred to as Hs14). All three datasets have been sequenced with high coverage Illumina paired-end (*i.e.*, PE-reads) and mate-pairs (*i.e.*, MP-reads) libraries. Moreover each organism has been assembled with up to 8 different assemblers.

GAGE provides high quality MP-libraries with narrow insert size distributions with standard deviation lower than 10% of the mean. However, narrow insert size libraries cannot be obtained in assembly projects where only small amounts of DNA are available. The MP libraries obtained in these cases are wide and the standard deviation can be up to 50% of the mean. BESST uses a technique that works well for larger uncertainties in insert size as this was one of the design assumptions. Therefore we have included the MP library provided in [22] which is characterised by a large variation in insert size. We used picard [23] to estimate the mean and standard deviation of insert size to 2600 and 1250 base pairs respectively. This library will from now on be referred to as the “wide MP” library. An insert size histogram of this distribution is available in Additional file 1: Figure S2.

### Evaluation

We scaffolded all 23 available (contig level) GAGE-assemblies with BESST v1.0.4.2, and the standalone scaffolders OPERA v1.2, SOPRA v1.4.6, and SSPACE-basic v2.0 using both PE and MP libraries provided by GAGE. Results for assembler-integrated scaffolders, as computed by GAGE, are also presented, but we primarily compare with the standalone scaffolders because they have access to the same amount of information as BESST and are applicable in similar situations. Note that in GAGE evaluation, Bambus2 was used both for contig and scaffold assembly (with unitigs provided by Celera Assembler).

All scaffolders were run with default parameters (see Additional file 1 for details) on a 1 TB RAM machine equipped with 24 CPUs. Read pairs were mapped to contigs using BWA v0.6.1 for BESST, OPERA, and SOPRA. SSPACE-basic is distributed with Bowtie, thus we used the included version of Bowtie (v0.12.5) for alignments with SSPACE. SSPACE also have a commercial version that supports alignments with BWA. The difference in read alignment method may have an impact on the scaffolding result but we did not investigate this. Out of the 124 scaffolding experiments, 117 successfully terminated within our runtime limit of 48 hours (OPERA and SOPRA were not able to scaffold the Hs14 dataset within this time limit in 3 and 4 cases respectively). Moreover, for the *Rhodobacter* genome, we also scaffolded the 8 available contig-level assemblies employing the wide MP library. To summarize, a total of 156 scaffolding experiments have been run, and of these, 149 terminated within the runtime limit and were evaluated.

Each of the 149 results have been evaluated with GAGE validation scripts http://gage.cbcb.umd.edu/results/gage-validation.tar.gzfor scaffolds, using the available reference sequence. For each assembly, we used GAGE evaluation scripts to compute:

-  Scaffold errors: number of indels, inversions, relocations, and translocations (as defined by [7]).
-  Scaffold NG50: size of the longest scaffold such that the sum of the lengths of all scaffolds longer than it is at least half of the (known) reference genome size.
-  Scaffold E-size: The expected scaffold size at a randomly chosen position on the genome (introduced and defined by [7]). The E-size is calculated as  where *L*_*c*_ is the length of scaffold *c* and *G* is the genome length estimated by the sum of all scaffold lengths. E-size is computed similarly for contigs.
-  Scaffold corrNG50: NG50 after scaffolds have been broken at every position a scaffold error is found.
-  Scaffold corrE-size: E-size after scaffolds have been broken at every position a scaffold error is found.

Moreover, for each entry, we also compute:

-  Number of initial contigs and number of produced scaffolds.
-  Time used by the scaffolder (without considering time required to align reads).

NG50 is a common metric to evaluate an assembly, often offering a good indication of the connectivity as it gives the weighted median contig length. However, the size of one scaffold can be misleading as a measure of the general connectivity of an assembly (as discussed in [7]) Consider, for example, a simple case of two error free assemblies *a* and *b* of a 1000 bp genome. If assembly *a* has one contig of 499 bp and 5 contigs of 100 bp while assembly *b* has 10 contigs of 100 bp, both will have an NG50 of 100 bp. The measure will therefore not expose the difference in quality between *a* and *b*. However, the respective E-sizes for assembly *a* and *b* are 299 and 100, and thus better capturing the average assembly fragmentation.

### Results

Tables 1, 2 and 3 presents the scaffolding performances for high quality libraries provided by GAGE. With the evaluation metrics provided here, no stand-alone scaffolder is a clear winner (as expected [7, 20]). In general, BESST produces favorable results on all of the organisms. Contrary to the results in [8], SOPRA does not perform well on the metrics provided by GAGE. The results for assembler-integrated scaffolders, as computed by GAGE, are presented alongside the stand-alone scaffolders results. There is a large variation in performance of integrated scaffolders but in general, BESST fares well also here. We note that only Allpaths-LG has better scaffolded assembly on all three GAGE datasets. Scaffolds from Bambus2 on *S. aureus* and SGA on Hs14 are two other instances where the integrated scaffolder outperforms the stand-alone ones.

**Table 1:** ***Staphylococcus aureus*** **GAGE data**

	BESST		OPERA		SOPRA		SSPACE		Integrated scaffolder		Unscaffolded
	**CorrEsize**	**err**	**CorrEsize (kbp)**	**err**	**CorrEsize (kbp)**	**err**	**CorrEsize (kbp)**	**err**	**CorrEsize (kbp)**	**err**	**CorrEsize (kbp)**
ABySS	263,4	**1**	**316,7**	12	103,4	2	126,3	5	35,3	1	31,4
Allpaths-LG	436,4	**0**	607,4	12	295,5	**0**	**1030,0**	1	1136,2	0	90,0
Bambus2	**827,3**	**1**	560,0	4	125,2	2	665,7	2	1119,5	0	19,6
MSR-CA	744,7	3	302,4	11	117,4	**0**	**781,6**	2	999,9	3	50,3
SGA	75,1	**0**	**920,1**	3	239,9	6	32,6	2	162,9	1	4,7
SOAPdenovo	**346,9**	**0**	333,1	7	227,2	**0**	286,7	5	229,3	0	68,0
Velvet	204,2	4	**236,8**	5	154,4	**1**	162,2	12	194,6	17	48,5
SUM	2898,1	**9**	**3276,6**	54	1263,0	11	3085,1	29			

The numbers in bold face style indicate the best corrected E-size and number of errors among the stand-alone scaffolders for each assembly.

**Table 2:** ***Rhodobacter sphaeroides*** **, GAGE data**

	BESST		OPERA		SOPRA		SSPACE		Integrated scaffolder		Unscaffolded
	**CorrEsize (kbp)**	**err**	**CorrEsize (kbp)**	**err**	**CorrEsize (kbp)**	**err**	**CorrEsize (kbp)**	**err**	**CorrEsize (kbp)**	**err**	**CorrEsize (kbp)**
ABySS	**70,2**	13	65,8	20	44,9	17	34,7	**4**	73,4	3	6,9
Allpaths-LG	**2005,7**	**0**	852,1	4	425,4	2	1271,9	1	2401,7	0	35,9
Bambus2	1426,0	4	1446,0	8	**1469,0**	3	789,9	**1**	1348,4	2	16,2
CABOG	**474,0**	**2**	362,6	7	293,4	**2**	419,1	4	211,3	5	21,5
MSR-CA	**1757,5**	3	573,5	8	138,2	**1**	1579,8	2	2001,1	5	21,6
SGA	100,5	6	**148,3**	**5**	105,7	41	44,9	9	48,0	1	3,2
SOAPdenovo	**1551,2**	**0**	841,5	7	1477,1	3	1500,6	3	687,6	0	18,6
Velvet	332,9	**2**	**336,1**	10	175,6	11	329,6	6	348,1	19	16,7
SUM	**7718,1**	**30**	4626,0	69	4129,2	80	5970,5	**30**			

The numbers in bold face style indicate the best corrected E-size and number of errors among the stand-alone scaffolders for each assembly.

**Table 3:** **Hs14, GAGE data**

	BESST		OPERA		SOPRA		SSPACE		Integrated scaffolder		Unscaffolded
	**CorrEsize (kbp)**	**err**	**CorrEsize (kbp)**	**err**	**CorrEsize (kbp)**	**err**	**CorrEsize (kbp)**	**err**	**CorrEsize (kbp)**	**err**	**CorrEsize (kbp)**
ABySS	**21,6**	**13**	15,8	200	-	-	15,3	47	2,8	9	3,1
Allpaths-LG	513,6	32	311,0	104	194,9	17	**559,0**	**22**	4652,3	45	27,1
Bambus2	88,2	**75**	61,7	331	-	-	**99,0**	109	157,6	143	6,3
CABOG	**421,9**	31	349,1	77	234,0	**19**	411,0	23	347,7	597	30,7
MSR-CA	51,3	**95**	-	-	-	-	**51,9**	146	111,9	1068	5,9
SGA	**57,2**	58	3,5	**39**	22,2	2253	24,8	42	89,9	19	3,7
SOAPdenovo	**94,1**	211	-	-	-	-	75,3	**205**	99,2	268	9,8
Velvet	35,7	**52**	-	-	**75,4**	734	22,6	140	26,6	9156	3,0
SUM	**1283,6**	**567**	741,0	751	526,5	3023	1259,0	734			

The numbers in bold face style indicate the best corrected E-size and number of errors among the stand-alone scaffolders for each assembly.

High quality datasets where insert size variation does not deviate much from the mean are not always available (see Additional file 1 for a discussion of this). The wide MP library has higher variation, and thus, increases the difficulty in scaffolding by introducing more uncertainty for contig placement. Table 4 shows scaffolding results for the wide MP library. Considering this scenario, BESST is outperforming the other stand-alone scaffolders having the highest total connectivity whilst giving the fewest errors. OPERA shows a slightly higher connectivity in some cases yet produces 17 times more errors in total. Withstanding the SGA assembly, SOPRA shows few errors in all cases. Yet in most cases, SOPRA also shows extremely low connectivity close to the original contig assembly. Similarly, SSPACE is shown to produce few errors but also struggle with connectivity. As mentioned, larger variation in library insert-size introduces more uncertainty of distance estimates and placement of contigs. Thus, scaffolding becomes more error prone. We believe that our performance here is a consequence of BESST’s ability to infer correct link-statistics despite wide library distribution.

**Table 4:** ***Rhodobacter sphaeroides*** **on GAGE contig assemblies using the wide MP library**

	BESST		OPERA		SOPRA		SSPACE		Unscaffolded
	**CorrEsize (kbp)**	**err**	**CorrEsize (kbp)**	**err**	**CorrEsize (kbp)**	**err**	**CorrEsize (kbp)**	**err**	**CorrEsize (kbp)**
ABySS	17,6	5	**19,7**	51	6,7	4	9,9	**1**	6,9
Allpaths-LG	**318,1**	**0**	314,5	1	44,4	**0**	70,3	2	35,9
Bambus2	**460,6**	**0**	267,1	0	93,9	**0**	137,9	**0**	16,2
CABOG	**199,8**	3	97,3	4	22,3	**0**	33,5	**0**	21,5
MSR-CA	192,1	1	**203,7**	6	24,0	**0**	38,1	1	21,6
SGA	4,3	**0**	**13,3**	111	3,3	12	6,5	3	3,2
SOAPdenovo	**756,7**	**0**	720,5	10	156,2	**0**	206,4	2	18,6
Velvet	**350,2**	**2**	62,3	4	17,8	3	30,9	3	16,7
SUM	**2299,3**	**11**	1698,4	187	368,7	19	533,6	12	

The numbers in bold face style indicate the best corrected E-size and number of errors among the stand-alone scaffolders for each assembly. Note that the corrected E-size for SOPRA is slightly less than the corrected contig size in the ABySS assembly. This can happen for low contiguity scaffolded assemblies that contains more bases than the contig assemblies (5,0Mbp and 4,5Mbp respectively on this instance). The difference in number of bases is due to the facts that GAGE evaluation script only compute statistics on contigs and scaffolds that are longer than 200bp. GAGE evaluation script returned an error when computing statistics for seven, two and one scaffolds on SSPACE results of ABySS, CABOG and MSR-CA assemblies respectively. On SOPRA and OPERA results, 1 respectively 3 scaffolds of the SGA assembly returned this error. We removed these scaffolds from the evaluation in order to compute the results. In all cases, the scaffolds removed summed up to a total length of less than 110 kbp. Thus, this has a negligible (either positive or negative) effect on E-size computation and an eventual positive effect on the number of errors. Results for BESST contained no scaffolds giving this error.

Tables 5, 6, 7 and 8 illustrates the types of errors that the stand-alone scaffolders make on the different data sets and Tables 9, 10, 11 and 12 shows runtime of the scaffolders excluding alignment time. An upper bound on runtime was set to 48 hours. BESST and SSPACE present good time scalability in contrast to SOPRA and OPERA which did not finish after 48 hours on 3 and 4 Hs14 instances respectively.

**Table 5:** **Types of errors on** ***S. aureus*** **summed over all assemblies**

Assembly	BESST	OPERA	SOPRA	SSPACE
Indels	2	17	2	6
Inversions	6	30	6	16
Translocations	0	0	0	1
Relocations	1	7	3	6

**Table 6:** **Types of errors on** ***Rhodobacter sphaeroides*** **summed over all assemblies**

Assembly	BESST	OPERA	SOPRA	SSPACE
Indels	16	28	13	7
Inversions	4	9	7	3
Translocations	2	26	21	14
Relocations	8	6	39	6

**Table 7:** **Types of errors on Hs14 summed over all assemblies**

Assembly	BESST	OPERA	SOPRA	SSPACE
Indels	398	149	1062	442
Inversions	163	383	154	289
Translocations	0	0	0	0
Relocations	6	219	1807	3

**Table 8:** **Types of errors on** ***Rhodobacter sphaeroides*** **with wide MP library summed over all assemblies**

Assembly	BESST	OPERA	SOPRA	SSPACE
Indels	5	87	4	3
Inversions	3	5	1	0
Translocations	1	9	1	7
Relocations	2	86	13	2

**Table 9:** **Runtime for scaffolders on** ***Staphylococcus aureus***

	Runtime (hh:mm:ss)			
**Assembly**	**BESST**	**OPERA**	**SOPRA**	**SSPACE**
ABySS	00:00:40	00:28:47	01:18:24	00:00:26
Allpaths	00:00:25	00:00:47	00:11:56	00:00:20
Bambus2	00:00:26	00:00:49	00:22:11	00:00:21
MSR-CA	00:00:26	00:01:05	00:19:21	00:00:21
SGA	00:01:03	00:05:58	04:30:08	00:00:51
SOAPdenovo	00:00:25	00:00:50	00:26:51	00:00:19
Velvet	00:00:27	00:00:53	00:39:25	00:00:21

**Table 10:** **Runtime for scaffolders on** ***Rhodobacter sphaeroides*** **with GAGE data**

	Runtime (hh:mm:ss)			
**Assembly**	**BESST**	**OPERA**	**SOPRA**	**SSPACE**
ABySS	00:01:22	00:12:38	01:17:49	00:00:40
Allpaths-LG	00:00:32	00:01:13	00:10:35	00:00:27
Bambus2	00:00:33	00:01:38	00:10:42	00:00:25
CABOG	00:00:35	00:00:59	00:11:13	00:00:27
MSR-CA	00:00:38	00:01:12	00:18:10	00:00:29
SGA	00:01:45	00:01:35	01:30:44	00:00:45
SOAPdenovo	00:00:33	00:03:48	00:18:08	00:00:27
Velvet	00:00:49	00:01:16	00:36:54	00:00:27

**Table 11:** **Runtime for scaffolders on Hs14 with GAGE data (upper bound time requirement was set to 48 hours)**

	Runtime (hh:mm:ss)			
**Assembly**	**BESST**	**OPERA**	**SOPRA**	**SSPACE**
ABySS	00:19:37	00:58:22	-	00:32:55
Allpaths-LG	00:05:06	00:53:24	22:02:09	00:12:55
Bambus2	00:07:43	01:18:06	-	00:14:26
CABOG	00:04:16	00:16:16	11:50:23	00:08:33
MSR-CA	00:11:22	-	-	00:15:38
SGA	00:53:42	00:23:18	16:15:22	00:38:42
SOAPdenovo	00:07:50	-	-	00:10:46
Velvet	00:10:07	-	01:27:16	00:15:35

**Table 12:** **Runtime for scaffolders on** ***Rhodobacter sphaeroides*** **with wide mate pair library**

	Runtime (hh:mm:ss)			
**Assembly**	**BESST**	**OPERA**	**SOPRA**	**SSPACE**
ABySS	00:00:52	00:04:33	00:14:49	00:00:50
Allpaths-LG	00:00:31	00:04:03	00:08:48	00:00:36
Bambus2	00:00:25	00:03:50	00:09:08	00:00:33
CABOG	00:00:31	00:03:10	00:07:17	00:00:37
MSR-CA	00:00:30	00:03:46	00:08:29	00:00:39
SGA	00:00:36	00:04:06	00:16:29	00:00:49
SOAPdenovo	00:00:27	00:04:35	00:09:54	00:00:35
Velvet	00:00:33	00:03:58	00:09:38	00:00:40

## Conclusion

We proposed a new algorithm, BESST, for the scaffolding problem. This algorithm works well on both small and large datasets. Moreover, we performed a large evaluation of our software against other popular stand-alone scaffolders. BESST places favorably compared to the other scaffolders on GAGE datasets and outperforms the other methods on libraries with a wide insert-size distribution.

## Methods

### Scaffolding of larger contigs

BESST works on a graph structure  (as defined under *Formalizing scaffolding* in the *Background* section). We apply statistics to assess similarity of observed link distribution to the expected link distribution between contigs larger than *μ*+4*σ*: a value chosen so that it is very unlikely that a properly mapped read pair will span over a contig of such size. This means that a correctly assembled contig that is not a perfect repeat will have at most one true edge to a neighboring contig of this size. However, the graph structure created for contigs of this size is in practice often far from linear due to *e.g.* small repeated regions and chimeric regions and that is why we want a way to assess edge quality.

The assessment of edges are realized in a score designed to reflect how reads from a read pair library should be placed on contigs if they were in fact correctly assembled close to each other on the genome. It consists of two parts, a link variation score *π*_*σ*_ and a link dispersity score *π*_*ζ*_, which we present in following subsections.

#### Link variation score (π_σ_):

Let *c*_*i*_,*c*_*j*_ be two correctly assembled contigs at distance *d* away from each other on the genome (with *d* small enough for the read-pair library to span). Reads linking *c*_*i*_ and *c*_*j*_ follow different distributions depending on the size of *d*, and a good estimate of *d* can be calculated using ML-estimation  with the tool GapEst introduced in [17]. Here, we go one step further and answer the question: Given *μ*,*σ* and  obtained from links observed over *c*_*i*_ and *c*_*j*_, what should the standard deviation of these links be? We denote this quantity with *σ*_*o*|*d*_ (standard deviation of observations given a gap size) to be consistent with the notation used for GapEst. Theorem 1 gives the theoretical expected value of *σ*_*o*|*d*_ which is dependent on the read length *r* and the length of the longer and shorter contig giving rise to the gap (denoted *c*_*min*_,*c*_*max*_).

**Theorem 1.***Given μ*,*σ and d*, *σ*_*o*|*d*_*is given by*

where *g* (*d*) and *q* (*d*) are defined in Additional file 1.

*Proof*. Derivation shown in Additional file 1.

We now define *π*_*σ*_ as


This quantity is a measure of how far observed distances are from the theoretical distance. Note that 0≤*π*_*σ*_≤1.

#### ***Link dispersity score (π***_***ζ***_***)***

The other part of the scoring function is an indicator of how well dispersed links are over the contigs they are connecting, given an estimated gap *d* between them. This dispersity is scored by the two sample Kolmogorov-Smirnov statistic [24] that gives a measure of difference in distribution between two independent samples. Letting observations on a contig be a sample, the independence between samples comes from the fact that the aligner has no information about which contigs lie close together, thus links between contigs can be seen as two independent alignment events.

**Figure 2:**
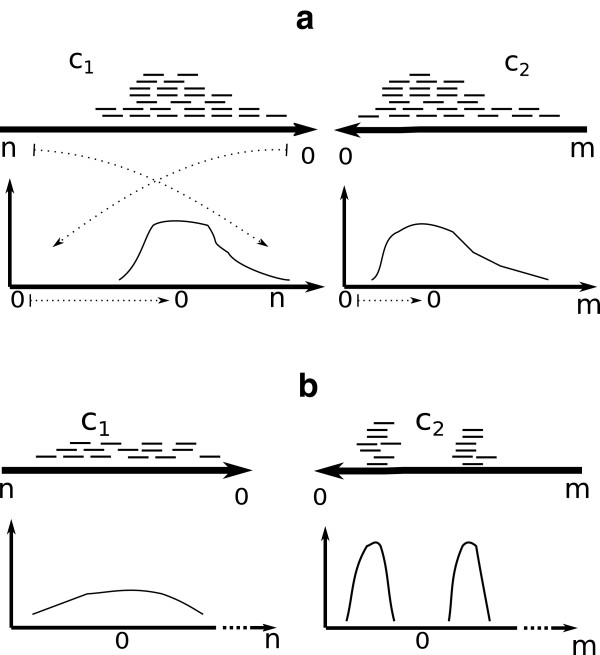
**Dispersity score.** Illustration of dispersity measurement. Read pairs linking contigs *c*_1_ and *c*_2_ of lengths n and m respectively are transformed to data tested with the KS-test. **(a)** Observations from contig *c*_1_ are translated and reflected on the x-axis while observations from contig *c*_2_ are translated. The two sample KS statistic will indicate high similarity in read distribution. **(b)** Strange placement of linked reads occur. Several explanations are possible. One possible explanation is that contig *c*_2_ is misassembled (chimeric) and another explanation is that *c*_2_ is a correctly assembled contig with small repeated regions solved on assembly level. The repeat might not be present in other contigs from the assembly and therefore, the alignments to these regions are reported as unique. Contig *c*_2_ is however not close to the to contig *c*_1_ on the genome and linked reads fail to place at the non-repeated regions on *c*_2_. The KS test will indicate low similarity

By observing if link distribution is similar for two linked contigs, we can detect abnormal edges that might come from one or several smaller repeats residing within a contig (see Figure 2b). We call this score the dispersity score and it is calculated as follows. Before testing, translation and reflection of the observations are necessary (see Figure 2a).

Let *n* read pairs link two contigs *c*_1_ and *c*_2_ where *c*_1_ is of length *m*. Recall that  are the *i*th observations on *c*_1_ and *c*_2_ respectively. Let ,  where  is the mean observation on *c*_1_ and similar for . Samples *y*^*i*^ are therefore the transformed observations as seen in Figure 2. The empirical distribution of samples is given by


where  is the indicator function. Let  and  be the two empirical distributions for the samples on *c*_1_ respectively *c*_2_, the two-sample KS statistic of the observations is then obtained by


It follows that *D*_*n*_∈ [0,1] since this quantity measures the largest distance between the two empirical cumulative distributions. The similarity score is defined as *π*_*ζ*_= 1 - *D*_*n*_.

#### Scoring edges:

To sum up the two previous subsections, we first derived a score for the ratio of the expected to the observed standard deviation of distance for links spanning a gap. Secondly, we gave a similarity score of expected to observed dispersity of links spanning a gap using the two-sample KS statistic. The total score of a gap-edge is defined as


By definition, we have 0 ≤ *π* ≤ 2. We have used the heuristic cutoff 0.5 which means that if any of the two quantities deviates more than twice from the assumed value, the score is set to 0, *i.e.*, the edge is discarded from the graph. *π* is only calculated on edges where *c*_*i*_,*c*_*j*_>*μ* + 4 *σ*. That is, any vertex that has more than 2 neighbors in this subgraph is considered to be involved in a region with linking errors since by the constraints of the contig lengths, the library should not be able to link more than one such contig. The score is used to choose the edge with links that best resemble the library. If the two highest scores in such a region are close to each other (their ratio higher than 0.9 set heuristically), we chose to not make a decision. This can for instance occur from larger repeated contigs. This approach finds a mapping *ϕ* on , representing a scaffolding, in  time.

#### Note about scoring edges

It might be inviting to start using *p*-values that can be estimated from the distributions we have defined. This would lead to a statistical test for keeping or discarding edges in the scaffold graph. However, this is not suitable for the problem in hand. Fewer links between contigs gives more uncertainty (leading to volatile *p*-values) and can lead to inability to discard many edges with low link support. Edges with many links would also be sensitive to smaller aberrations by increased sensitivity of statistical testing with larger sample sizes. In the case of multiple edges from a contig, we want to compare edges to see which observations have matching distributions. Comparing significance levels of *p*-values to make this decision is bad practice since *p*-values are nonlinear transforms of data that should only be interpreted under the null hypothesis. That is, the *p*-value is a measure of evidence; it is not an estimate of effect size. Looking at the similarity ratio for *π*_*σ*_ and the KS statistic for *π*_*s*_ provides a measure that is robust to the number of links and can be used to measure the fit of data when a decision is needed.

### Including smaller contigs

Small contigs are defined as having a length less than *μ* + 4 *σ*, *i.e.*, all contigs not treated in the previous section. This limit varies with respect to the current library and is used to efficiently create linear scaffolds (as explained in previous section). There are limitations when scaffolding with contigs of size over a particular threshold. Firstly, one will have gaps in these scaffolds where shorter contigs could be placed. Secondly, several small contigs can occur between two large scaffolds making read pairs unable to link them together. We address this issue as follows.

In graph theory, a *simple* path in  is a path without repeated vertices. Let a *connection* between two large contigs *c*_*a*_ and *c*_*b*_ in  be defined as a simple path starting at *c*_*a*_ and ending at *c*_*b*_ with the rest of its vertices as small contigs. For a connection *γ* consisting of a sequence of *n* contigs {*c*_1_,…,*c*_*n*_}, we define *g*(*c*_*i*_) to be the number of links that goes from *c*_*i*_ to any other contig *c*_*j*_∈ *γ*. Similarly, let *b*(*c*_*i*_) be the number of links that go from *c*_*i*_ to any other contig *c*_*j*_∉ *γ*. The notation of *g* and *b* are chosen to indicate “good” links and “bad” links respectively, for a given connection *γ*. The score of *γ* is defined as


**Figure 3:**
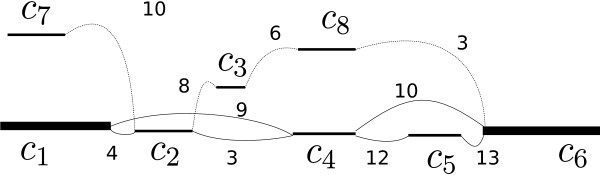
**Small contigs.** An example of a region in  containing smaller contigs. There are 5 possible paths to connect *c*_1_ and *c*_6_. The highest scoring one is [*c*_1_,*c*_4_,*c*_5_,*c*_6_], with , giving *π*_*p*_= 34 and it is the selected path between *c*_1_ and *c*_6_. In this path the good edges are represented as solid lines and bad edges are represented as dotted lines. A lower-score alternative is [*c*_1_,*c*_2_,*c*_4_,*c*_5_,*c*_6_] with (*g*(*c*_*i*_),*b*(*c*_*i*_))=(47,18), giving *π*_*p*_= 29. *c*_2_ is a problematic contig that can be chimeric or consists of repeated sequence(s). The three remaining paths, all of them with negative score, are [*c*_1_,*c*_2_,*c*_3_,*c*_8_,*c*_6_],[*c*_1_,*c*_4_,*c*_6_],[*c*_1_,*c*_2_,*c*_4_,*c*_6_].

*i.e.*, the sum of differences of good and bad links for all contigs belonging in *γ*. An example region of connections is shown in Figure 3. To find connections between two large contigs *c*_*a*_,*c*_*b*_, we use breadth-first search in . If more than one connection is found, the highest scoring one is chosen if the score is positive.

If *c*_*a*_ and *c*_*b*_ is within an already created scaffold, the algorithm will look for paths with length less than *d* + 2 *σ*, where 2 *σ* allows for uncertainty of the estimate of *d*. If *c*_*a*_ and *c*_*b*_ are not within an already created scaffold, there is no distance constraint on the length of the connection. For dense regions in , there can be an exponential blow-up in the number of possible connections. We have set a threshold limiting the breadth-first search to 1000 iterations. This restricting threshold is motivated by two reasons. Firstly, dense regions are likely to be caused by spurious edges. Thus, paths created in these regions will often have a negative score. Secondly, we have seen from large datasets that correct connections tend to be short. This makes higher ordered layers in the breadth first search contain connections with negative score or paths that does not lead to a valid end vertex (to form a connection).

All connections with a positive score are used to improve the contiguity of the scaffolds. First, gaps within existing scaffolds are filled if a connection with a positive score has been found. In a second step, connections between scaffolds are considered. The extension starts with the highest scoring connection first and proceeds in a descending order of the score. If a contig is found in multiple connections with positive scores, it is only used in the one with the highest score.

### Using multiple libraries

If given multiple libraries, BESST uses these libraries in an increasing order of library insert size. Scaffolds created in earlier steps are seen as contigs for the next library.

### Implementation

BESST source code is available under the GNU GPL v3 license. It is implemented in Python using Networkx [25] graph library to represent the scaffolding graph and pysam [26] for parsing BAM files. As input BESST takes contigs in a FASTA file and the alignments of the paired reads to the contigs as sorted BAM files. BESST can use several paired-read libraries with different insert sizes. The main output consists of scaffolds in a FASTA file. If several libraries are used, a scaffold FASTA file is given as output in each scaffolding step. The output also consists of AGP- and GFF-files that contain information about the scaffolds, such as position of each contig in the scaffold and length of the gaps. The contigs that were classified as repeats are output in a separate FASTA file.

#### Preprocessing of 

When initializing , BESST computes different statistics of the read library such as mean and standard deviation of insert size (*μ*,*σ*) and of the coverage (*μ*_*c*_,*σ*_*c*_). Links with inconsistent insert size defined as *o*_1_+ *o*_2_>*μ* + *lσ* are not considered in the scaffolding since they are likely to be placed on chimeric contigs or misaligned. Here, *l* is a user defined constant which defaults to 6.

BESST removes the contigs that, based on coverage, behave as repeats. A contig *c*_*i*_ is classified as a repeat if coverage of *c*_*i*_ is larger than max {2 *μ*_*c*_,*μ*_*c*_+ *tσ*_*c*_}, where *t* is calculated with the same principle as computing *k* for *π*_*ζ*_.

#### Data and optional parameters as input to BESST

BESST uses alignments of paired reads to contigs in format of sorted BAM files. A read aligner such as BWA or Bowtie [27, 28] can be used to map the paired reads in forward reverse mode. We use those read pairs whose both ends map to a unique position in the collection of contigs.

Several parameters for BESST can optionally be set on the command line:

-  *μ* and *σ* can be specified instead of being computed internally. This can be good if the assembly is very fragmented.
-  Minimum number of links needed to create an edge (with 5 as default value).
-  Coverage cutoff for repeat identification.
-  Duplicate read remover (based on identical map positions of both fragments in a paired read).
-  The inclusion of small contigs can be inactivated.

## Availability

Implementation of BESST is provided at https://pypi.python.org/pypi/BESST and https://github.com/ksahlin/BESST.

## Electronic supplementary material

Additional file 1: **Supplementary Material.** Document with supplemental information. This document contains additional evaluation results and proof of Theorem 1. (PDF 545 KB)
