# Supplementary material for: BESST - Efficient scaffolding of large fragmented assemblies
Source: BMC Bioinformatics. 2014 Aug 15;15(1):281. doi: 10.1186/1471-2105-15-281 (PMC4262078; doi:10.1186/1471-2105-15-281)
Supplement: Supplementary file 1 — Additional file 1: Supplementary Material. Document with supplemental information. This document contains additional evaluation results and proof of Theorem 1. (PDF 545 KB) [file 12859_2014_6686_MOESM1_ESM.pdf]

BESST: Efficient scaffolding of large fragmented assemblies.

Supplementary Material

July 22, 2014

Contents

1 Evaluation **1**
- 1.1 About evaluation . . . 1
- 1.2 Mate-pair distribution bias . . . 2
- 1.3 Experiments set up . . . 3
- 1.4 Supplementary Results . . . 6
- 1.5 run commands . . . 7
  - 1.5.1 BWA . . . 7
  - 1.5.2 BESST . . . 7
  - 1.5.3 Opera . . . 7
  - 1.5.4 SOPRA . . . 7
  - 1.5.5 SSPACE . . . 8
- 1.6 Supplementary Figures . . . 8
- 1.7 Tables . . . 12
  - 1.7.1 Staphylococcus . . . 12
  - 1.7.2 Rhodobacter . . . 14
  - 1.7.3 Human chromosome 14 . . . 16

2 Proof of Theorem 1 **18**
- 2.1 Function definitions . . . 18
- 2.2 Derivation . . . 18

1 Evaluation

1.1 About evaluation

De novo assembly validation is a task as difficult as assembly itself. All recent evaluation efforts conclude that, in general, assembler performance is deeply correlated to the dataset being assembled. GAGE [4] showed how the same assembler can have very different results and performance even on similar genomes. Moreover, GAGE [4] as well as Assemblathon 1 and 2 [2] demonstrated how difficult *de novo* assembly evaluation can be. As an example, consider that the

Assemblathon 2 conclusion was not made available until after a year. In general, the take-home-message of all previously cited studies is the following:

(i) no single metric is able to fully and easily describe assembly quality and correctness;

(ii) the most used length-based statistics like N50 and NG50 (given a set of contigs, each with its own length, the N50 length is defined as the longest length for which the collection of all contigs of that length or longer contains at least half of the total assembly length, the definition is similar for NG50, in this case the total assembly length is replaced by the estimated assembly length) and total assembly length are *bad* quality predictor and cannot be used to deduce the overall assembly quality (refer to [5, 6] for a detailed discussion);

(ii) even in presence of a high quality reference sequence *de novo* assembly evaluation is an extremely difficult task (see GAGE and Assemblathon 1 cases);

(iv) the same tool employed on a different dataset (*i.e.*, a different genome, different libraries, different sequencers, *etc.*) can produce utterly different results.

In the last 5 years more than 20 assemblers have been published. More often than not, new tools proved their own superiority with then existing ones by showing better performances (usually contiguity based statistics) on a single or few datasets. The situation holds true also in the case of scaffolders: not only is scaffolding performance typically evaluated using bad quality predictors (*e.g.*, NG50), but, to the best of our knowledge, nobody as explored how contigs produced by different assemblers may or may not influence stand-alone scaffolder performance.

Conscious of the current limitations in assembly evaluation we tried to perform an evaluation of our tool as unbiased as possible and able to provide to the final users a complete picture of the advantages of BESST over other stand-alone scaffolders.

No single metric is able to fully or easily describe the quality of an assembler/scaffolder. For this reason we computed several metrics. In particular we computed length-based statistics (NG50, Number of Scaffolds), and, thanks to the availability of a reference sequence, reference-based statistics (corrected NG50, and number of mis-assemblies). For each tool, we computed the required run-time (clearly the speed of a tool does not suggests nothing about the quality, however a user must know if the tool he wants to use can at least provide an output in feasible time).

1.2 Mate-pair distribution bias

In the main manuscript we emphasize the fact that BESST performs better than other standalone scaffolders in presence of wide (i.e., high standard deviation)

insert sizes distributions. Generating mate-pair (MP) libraries with a tight insert size variation (e.g., 10% of the expected and/or observed mean insert size) have been a difficult step in many early de novo assembly projects based on Illumina technology (see [1]). Recently, the introduction of the new Nextera Mate Pair protocol allows all labs to produce good MP libraries. However, this is true only if there is enough DNA available (i.e., 4 or more micro-grams) during library preparation. When DNA is scarce, gel cut selection cannot be performed, therefore yielding a much wider insert size distribution. In Figure S1 we show the insert size distribution plot of two Nextera Mate Pair Libraries: Figure S1a has been obtained starting from 5 micrograms of DNA, thus applying the gel-cut step. Whereas the library plotted in Figure S1b has been obtained starting from 1 microgram of DNA, therefore without using the gel-cut procedure. Both samples are sequenced from a human genome and aligned against the human genome reference Hg19. Distribution plots have been obtained with Picard tools. Insert size distributions characterized by a large standard deviation are common also when trying to obtain large inserts (¿ 8 kbp). Therefore, there are still many situations where a narrow insert size distribution is not possible to obtain.

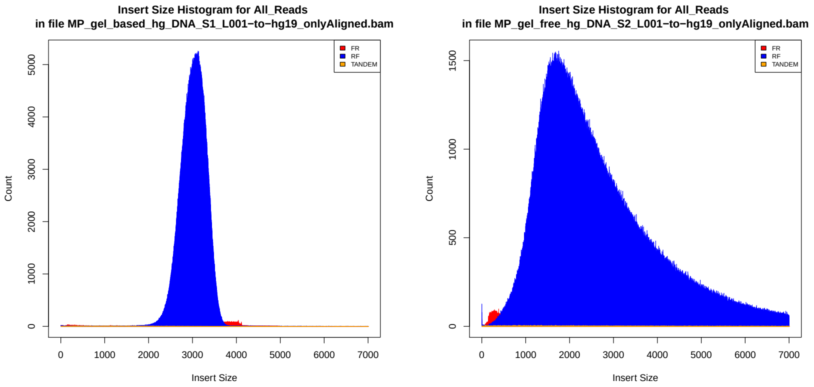

(a) Nextera Mate Pair with gel cut (starting DNA material of 5 micrograms)

(b) Nextera Mate Pair without gel cut (starting DNA material of 1 micrograms)

Figure S1: Insert size distribution of Mate Pairs with two variants of Nextera Mate Pair Kit. RF: reverse-forward orientated reads. FR: forward-reverse orientated reads. Tandem: forward-forward or reverse-revers oriented reads.

1.3 Experiments set up

GAGE [2] is the study that, so far, offers the best datasets to test a new software. Datasets for three highly different organisms togheter with finished reference sequences are provided. Each of the organisms have in turn been assembled

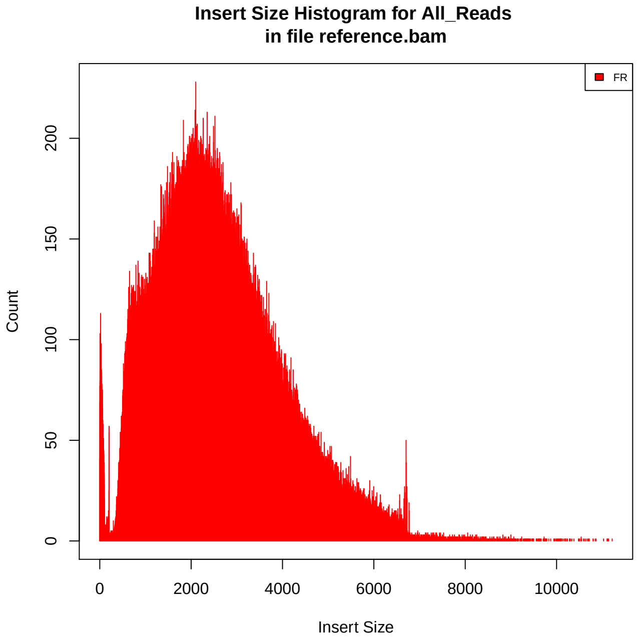

Figure S2: Insert size distribution whide mate pair distribution from Rhodobacter s.

with 8 different assemblers. Scripts to evaluate (but not rank) the assemblies are also provided to the community.

The three GAGE datasets are *Staphylococcus aureus* (genome size 2.872.915), *Rhodobacter sphaeroides* (genome size 4.603.060), and Human chromosome 14 or Hc14 (ungapped size 88.289.540). All three datasets consists of two libraries: one paired-end library (average insert size 200 bp) and one mate-pair library (average insert size 3 Kbp). GAGE provide assemblies (both contigs and scaffolds) obtained with 8 different assemblers (ABySS, ALLPATHS-LG, Bambus2, CABOG, MSR-CA, SGA, SOAPdenovo, Velvet), however only 7 are available for *Staphilococcus a.* dataset as CABOG failed. Such assemblies can reasonably be considered the best achievable assemblies, as they were obtained by *de novo* assembly experts.

Our analysis and evaluation has been performed on the three original GAGE dataset, plus one fourth partially simulated dataset. This last dataset has been partially simulated in order to show how libraries characterized by insert sizes with large standard deviations (*e.g.*, many of the mate-pair library produced) badly affect the majority of scaffolders. In particular, a mate-pair-like library characterized by a mean insert size of 3 Kbp and an insert standard deviation of 1.5 Kbp has been simulated using *Rhodobacter s.* reference sequence. Such sim-

ulated library provided a raw coverage of 30×. This simulated library, together with the original pair-end library has been used to scaffold all *Rhodobacter s.* contig-level assemblies.

For each available GAGE assembly (in contig version) we used 4 different stand-alone scaffolders:

- OPERA v1.2
- SOPRA 1.4.6
- SSPACE 2.0
- BESST 1.0.4.2

All scaffolders have been run with default parameters, see section 1.5 for details. The script that automatically run all the scaffolders and perform the reference-based evaluation is available at `http://gage.cbcb.umd.edu/results/index.html` (note, the script need a large number of programs like samtools, mummer to be available in the main path).

To summarize we run a total of 124 scaffolding experiments on the original GAGE datasets. However, "only" 117 have been evaluated as SOPRA and OPERA did not finish in time on 3 and 4 Hs14 instances respectively. In particular, SOPRA and OPERA were not able to provide output after 48 hours.

All experiments have been run on a 1TB RAM machine equipped with 24 CPU. All scaffolders have been run using a single CPU. Even though we employed such a powerful machine, none of the tested scaffolders required an unreasonable amount of RAM memory.

For each of 117 successfully completed scaffolding experiments we used the GAGE evaluation script to compute:

- NG50: size of the longest scaffold such that the sum of the lengths of all scaffolds longer than it is at least half of the genome size;
- corrNG50: original assembly is break every time a mis-assemble is found. corrNG50 is the NG50 computed on this set of "error-free" scaffolds;
- mis-assemblies: number of inversions, relocations, and translocations identified by `getScaffoldStats` script found on GAGE homepage.

Moreover, for each entry, we also compute:

- number of initial contigs and number of produced scaffolds;
- time required by the scaffolder (without considering time required to align reads).

1.4 Supplementary Results

Figures S3-S5 and Tables S2-S24 summarizes all experiments performed. All results are reported with the only exception of S5c where the x-axis have been trimmed to 450 (*i.e.*, 450 errors) in order to allow the visualization of the other results. The only two data points that are excluded are velvet and sga scaffolded with SOPRA that have 734 and 2253 misassemblies respectively.

Figures S3 and S4 show the relationship between the ratio between corrLG50 and LG50 and the ratio between the number of scaffolds and the number of original contigs. The former Figure summarizes the result for all the datasets (symbols represent scaffolders, colors represent assemblers, and symbol sizes represents the genomes, *i.e.*. symbols size is proportional to genome size). The latter Figure is divided into three sub-figures (Figures S4a, S4b, and S4c) and represents the same data of Figure S3 but for one dataset (*i.e.*, genome) per time. From these figures it is clear that there is no winner, *i.e.*, there is no assembler always performing better than others. In general, looking also at Tables S2-S24, BESST and SSPACE achieve good results, however both tools have some outliers. As an example, consider Hc14 dataset on ABySS assembly (big red square and cross in the right bottom corner of Figures S3 and S4c). On this specific dataset all scaffolder perform badly not being able to scaffold the highly fragmented contigs produced by ABySS.

Figure S5 summarize for each dataset (the three original GAGE dataset, Figures S5a-S5c plus the one partially simulated dataset, Figure S5d) the relationship between number of mis-assemblies (*i.e.*, x-axis) and corrected LG50 (*i.e.*, y-axis).
Figures S5a-S5c clearly highlight the good performances of BESST and SSPACE over OPERA and SOPRA. Such behaviour is even more evident in Figure S5d. SOPRA assemblies form a cloud in the bottom left corner (*i.e.*, few errors but extremely fragmented assembly), while OPERA is almost always the scaffolder introducing the highest number of mis-assemblies.

By comparing Figures S5b and Figure S5d we appreciate how BESST is less affected by the large insert size variation. When scaffolding ABySS, BAMBUS, CABOG, MSR-CA, SGA, SOAPdenovo, and Velvet contigs, BESST is not affected by the large variation, while SSPACE is badly affected (as an example, in MSR-CA case, corrNG50 decreases from 2.5 Mbp down to 1 Mbp). In in ABySS case, both scaffolders are unable to increase corrNG50. When working with Allpaths-LG contigs both BESST and SSPACE improve with BESST always being the one characterized by the lowest amount of mis-assemblies (*i.e.*, 0).

1.5 run commands

For scaffolders requiring specification of insert size of library. these values were provided

organism	library	mean	sd
S. aureus	short frag	180	30
S. aureus	long frag	3500	300
Rhodo	short frag	180	30
Rhodo	long frag	3500	300
Hs14	short frag	180	30
Hs14	long frag	2600	300
Rhodo	wide MP	2600	1250

Table S1: insert size specification

1.5.1 BWA

1.5.2 BESST

Assuming BESST is installed with pip or easy_install (other wise run BESST from directory where "runBESST" exists). BESST has automated inference of library distribution.

```
$ runBESST -c /path/to/contigs.fasta -f /path/to/BAM_file -o /path/to/output
```

1.5.3 Opera

Assuming opera is in your system path

```
$ perl /path/to/operafolder/opera_v1.2/bin/preprocess_reads.pl
/path/to/contigs /path/to/reads1 /path/to/reads2
/path/to/outdir/<mapped.bam> "bwa"

$ opera  /path/to/contigs  /path/to/outdir/mapped.bam /path/to/outdir
```

1.5.4 SOPRA

From SOPRA, stand alone scaffolder directory, *i.e.*

```
/path/to/SOPRA/source_codes_v1.4.6/SOPRA_with_prebuilt_contigs/
```

Paired reads needs to be interleaved into a single reads.fasta file

```
$ perl s_prep_contigAseq_v1.4.6 -contig /path/to/contigs
 -mate /path/to/reads.fasta -a /path/to/outdir
```

```
bwa index  -p <prefix> /path/to/outdir/contigs_sopra.fasta
```

```
bwa aln  -t 8 /path/to/outdir/contigs_sopra.fasta
/path/to/outdir/reads.fasta -f align_frag.sai

bwa samse contigs_sopra.fasta align_frag.sai
 frag_i_sopra.fasta > align_frag.sam

$ perl s_parse_sam_v1.4.6 -sam align_frag.sam
 -a /path/to/SOPRA_out

$ perl s_read_parsed_sam_v1.4.6 -parsed align_frag.sam_parsed
-d <insert size> -a /path/to/SOPRA_out

$ perl s_scaf_v1.4.6.pl -o /path/to/SOPRA_out
 orientdistinfo_c* -a /path/to/SOPRA_out
```

1.5.5 SSPACE

library.txt is a text file containing information about the libraries in the following format

```
lib1 /path/to/short_frag1.fa /path/to/short_frag2.fa <insert> 0.25 FR
lib2 /path/to/short_frag1.fa /path/to/short_frag2.fa <insert> 0.4 RF
```

(for the wide rhodo MP library, we specified 0.95 as the allowed error)

```
SSPACE -l library.txt -s /path/to/contigs -b /path/to/sspace_output
```

1.6 Supplementary Figures

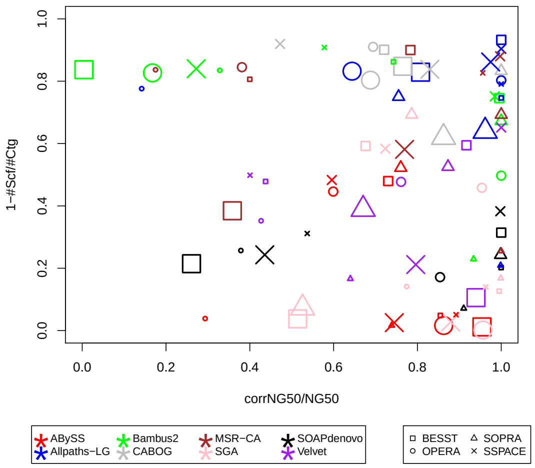

Figure S3: Corrected NG50 over NG50 versus 1 - (#Scaffolds/#Contigs) for all original GAGE datasets

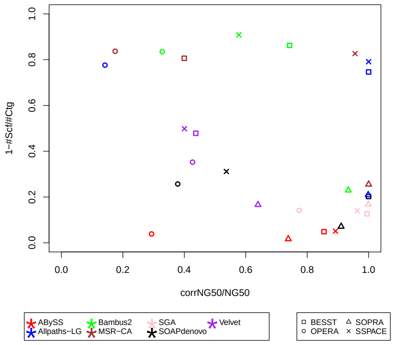

(a) Staphylococcus original GAGE dataset

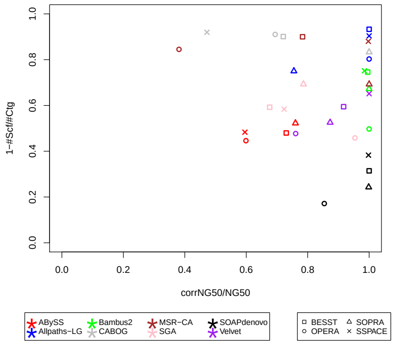

(b) Rhodobacter original GAGE dataset

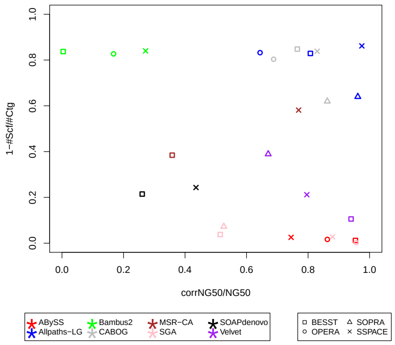

(c) Hc14 original GAGE dataset

Figure S4: Corrected NG50 over NG50 versus 1 - (#Scaffolds/#Contigs) for each distinct GAGE dataset.

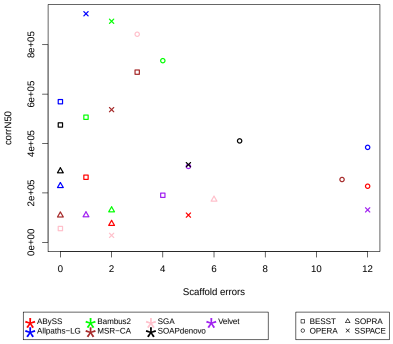

(a) Staphylococcus original GAGE dataset

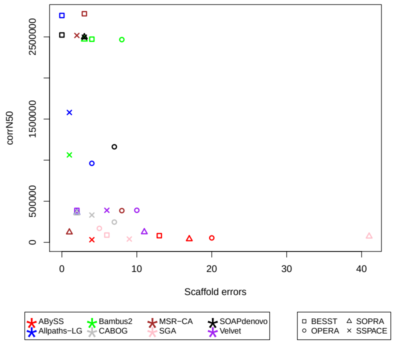

(b) Rhodobacter original GAGE dataset

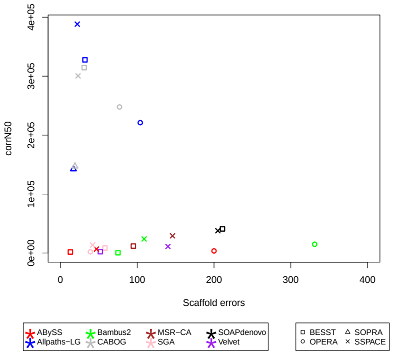

(c) Hc14 original GAGE dataset

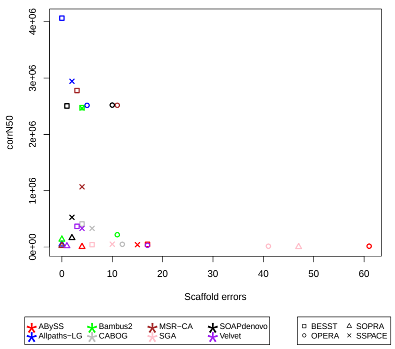

(d) Rhodobacter partially simulated dataset

Figure S5: Misassemblies versus corrected NG50 for each distinct GAGE dataset plus the partial simulated Rhodobacter dataset.

1.7 Tables

1.7.1 Staphylococcus

ABySS (contigs 5078)

Scaffolder	Scaffolds	NG50	Corrected NG50	Corrected E-size	MisAssemblies	Time (hh:mm:ss)
ABySS	5012	32467	27695	35318	1	-
BESST	4831	308307	263492	263432	1	0:00:40
OPERA	4882	774171	227151	316711	12	0:28:47
SOPRA	4995	101902	75253	103376	2	1:18:24
SSPACE	4921	123414	110108	126319	5	0:00:26

Table S2: Scaffolders evaluation: ABySS

ALLPATHS-LG (contigs 67)

Scaffolder	Scaffolds	NG50	Corrected NG50	Corrected E-size	MisAssemblies	Time (hh:mm:ss)
ALLPATHS-LG	19	1091731	1091731	1136265	0	-
BESST	17	569382	569339	436386	0	0:00:25
OPERA	15	2709269	384485	607356	12	0:00:47
SOPRA	53	228836	228836	295519	0	0:11:56
SSPACE	18	925399	925399	1029894	1	0:00:21

Table S3: Scaffolders evaluation: ALLPATHS-LG

Bambus2 (contigs 109)

Scaffolder	Scaffolds	NG50	Corrected NG50	Corrected E-size	MisAssemblies	Time (hh:mm:ss)
Bambus2	17	1083792	1083765	1119480	0	-
BESST	15	681784	506421	827277	1	0:00:26
OPERA	18	2241156	735458	559989	4	0:00:49
SOPRA	84	139423	130193	125238	2	0:22:11
SSPACE	13	1548961	894963	665707	2	0:00:21

Table S4: Scaffolders evaluation: Bambus2

MSR-CA (contigs 98)						
Scaffolder	Scaffolds	NG50	Corrected NG50	Corrected E-size	MisAssemblies	Time (hh:mm:ss)
MSR-CA	17	2411914	1021905	999871	3	-
BESST	19	1722534	688931	744749	3	0:00:26
OPERA	16	1453280	254304	302424	11	0:01:05
SOPRA	73	109325	109325	117403	0	0:19:21
SSPACE	22	561933	537139	781608	2	0:00:21

Table S5: Scaffolders evaluation: MSR-CA

SGA (contigs 6854)						
Scaffolder	Scaffolds	NG50	Corrected NG50	Corrected E-size	MisAssemblies	Time (hh:mm:ss)
SGA	546	208181	208181	162894	1	-
BESST	5986	56087	55805	75121	0	0:01:03
OPERA	5885	1087902	842455	920147	3	0:05:58
SOPRA	5703	173605	173441	239918	6	4:30:08
SSPACE	6176	29260	28179	32664	2	4:30:08

Table S6: Scaffolders evaluation: SGA

SOAPdenovo (contigs 183)						
Scaffolder	Scaffolds	NG50	Corrected NG50	Corrected E-size	MisAssemblies	Time (hh:mm:ss)
SOAPdenovo	175	331598	288182	229282	0	-
BESST	146	475286	475155	346863	0	0:00:25
OPERA	136	1084275	410614	333131	7	0:00:50
SOPRA	170	316522	288182	227222	0	0:26:51
SSPACE	125	585203	314255	286715	5	0:00:19

Table S7: Scaffolders evaluation: SOAPdenovo

Velvet (contigs 301)						
Scaffolder	Scaffolds	NG50	Corrected NG50	Corrected E-size	MisAssemblies	Time (hh:mm:ss)
Velvet	173	762333	126167	194624	17	-
BESST	157	435359	190483	204265	4	0:00:27
OPERA	195	719715	307250	236809	5	0:00:53
SOPRA	251	172158	110171	154422	1	0:39:25
SSPACE	175	327881	131323	162203	12	0:39:25

Table S8: Scaffolders evaluation: Velvet

1.7.2 Rhodobacter

ABySS (contigs 2966)						
Scaffolder	Scaffolds	NG50	Corrected NG50	Corrected E-size	MisAssemblies	Time (hh:mm:ss)
ABYSS	2714	7495	4590	7343	3	-
BESST	1543	110816	80918	70219	13	0:01:22
OPERA	1643	89771	53775	65797	20	0:12:38
SOPRA	1417	52888	40204	44907	17	1:17:49
SSPACE	1533	53255	31724	34738	4	0:00:40

Table S9: Scaffolders evaluation: ABySS

ALLPATHS-LG (contigs 208)						
Scaffolder	Scaffolds	NG50	Corrected NG50	Corrected E-size	MisAssemblies	Time (hh:mm:ss)
ALLPATHS-LG	38	3192334	3192322	2401705	0	-
BESST	14	2760566	2760566	2005741	0	0:00:32
OPERA	41	961305	961268	852138	4	0:01:13
SOPRA	52	478192	361053	425352	2	0:10:35
SSPACE	20	1580192	1580191	1271892	1	0:00:27

Table S10: Scaffolders evaluation: ALLPATHS-LG

Bambus2 (contigs 177)						
Scaffolder	Scaffolds	NG50	Corrected NG50	Corrected E-size	MisAssemblies	Time (hh:mm:ss)
Bambus2	92	2438508	2418550	1348427	2	-
BESST	45	2483791	2472107	1426037	4	0:00:33
OPERA	89	2467405	2467110	1446078	8	0:01:38
SOPRA	58	2471051	2470486	1468974	3	0:10:42
SSPACE	44	1080123	1063541	789938	1	0:00:25

Table S11: Scaffolders evaluation: Bambus2

CABOG (contigs 323)						
Scaffolder	Scaffolds	NG50	Corrected NG50	Corrected E-size	MisAssemblies	Time (hh:mm:ss)
CABOG	130	245073	55312	211290	5	-
BESST	32	500927	360973	473965	2	0:00:35
OPERA	29	354859	246350	362618	7	0:00:59
SOPRA	54	355986	355981	293405	2	0:11:13
SSPACE	26	702351	331664	419082	4	0:00:27

Table S12: Scaffolders evaluation: CABOG

MSR-CA (contigs 400)						
Scaffolder	Scaffolds	NG50	Corrected NG50	Corrected E-size	MisAssemblies	Time (hh:mm:ss)
MSR-CA	44	2975504	2967689	2001133	5	-
BESST	40	3551562	2781837	1757493	3	0:00:38
OPERA	62	1012116	385797	573494	8	0:01:12
SOPRA	123	122714	122714	138163	1	0:18:10
SSPACE	48	2524124	2517447	1579840	2	0:00:29

Table S13: Scaffolders evaluation: MSR-CA

SGA (contigs 4280)						
Scaffolder	Scaffolds	NG50	Corrected NG50	Corrected E-size	MisAssemblies	Time (hh:mm:ss)
SGA	2096	51880	51191	47965	1	-
BESST	1745	130172	88029	100532	6	0:01:45
OPERA	2319	177684	169478	148274	5	0:01:35
SOPRA	1314	92351	72614	105746	41	1:30:44
SSPACE	1782	54994	39781	44879	9	0:00:45

Table S14: Scaffolders evaluation: SGA

SOAPdenovo (contigs 350)						
Scaffolder	Scaffolds	NG50	Corrected NG50	Corrected E-size	MisAssemblies	Time (hh:mm:ss)
SOAPdenovo	312	660164	660070	687612	0	-
BESST	240	2523252	2523215	1551250	0	0:00:33
OPERA	290	1361068	1162393	841476	7	0:03:48
SOPRA	265	2501568	2497760	1477121	3	0:18:08
SSPACE	216	2501138	2495726	1500559	3	0:00:27

Table S15: Scaffolders evaluation: SOAPdenovo

Velvet (contigs 809)						
Scaffolder	Scaffolds	NG50	Corrected NG50	Corrected E-size	MisAssemblies	Time (hh:mm:ss)
Velvet	382	353027	270086	348058	19	-
BESST	328	423527	388338	332899	2	0:00:49
OPERA	423	512247	389760	336138	10	0:01:16
SOPRA	384	143316	125110	175580	11	0:36:54
SSPACE	282	389480	389480	329615	6	0:00:27

Table S16: Scaffolders evaluation: Velvet

1.7.3 Human chromosome 14

ABySS (contigs 900934)						
Scaffolder	Scaffolds	NG50	Corrected NG50	Corrected E-size	MisAssemblies	Time (hh:mm:ss)
ABySS	900081	1274	1301	2772	9	-
BESST	890267	1803	1720	21555	13	0:19:37
OPERA	886073	4135	3567	15833	200	0:58:22
SOPRA	-	-	-	-	-	
SSPACE	878095	8942	6660	15322	47	0:32:55

Table S17: Scaffolders evaluation: ABySS

ALLPATHS-LG (contigs 4722)						
Scaffolder	Scaffolds	NG50	Corrected NG50	Corrected E-size	MisAssemblies	Time (hh:mm:ss)
ALLPATHS-LG	418	81646936	3471817	4652301	45	-
BESST	808	405829	327693	513648	32	0:05:06
OPERA	792	343486	221185	310988	104	0:53:24
SOPRA	1702	147969	142295	194879	17	22:02:09
SSPACE	653	398276	388209	558947	22	0:12:55

Table S18: Scaffolders evaluation: ALLPATHS-LG

Bambus2 (contigs 13593)						
Scaffolder	Scaffolds	NG50	Corrected NG50	Corrected E-size	MisAssemblies	Time (hh:mm:ss)
Bambus2	1792	369868	100528	157597	143	-
BESST	2214	112697	446	88198	75	0:07:43
OPERA	2352	88972	14926	61689	331	1:18:06
SOPRA	-	-	-	-	-	-
SSPACE	2174	87839	23882	98968	109	0:14:26

Table S19: Scaffolders evaluation: Bambus2

CABOG (contigs 3451)						
Scaffolder	Scaffolds	NG50	Corrected NG50	Corrected E-size	MisAssemblies	Time (hh:mm:ss)
CABOG	498	401279	242612	347722	597	-
BESST	539	411044	314460	421903	31	0:04:16
OPERA	696	360144	247795	349075	77	0:16:16
SOPRA	1347	170582	147102	234039	19	11:50:23
SSPACE	573	362125	300452	410970	23	0:08:33

Table S20: Scaffolders evaluation: CABOG

MSR-CA (contigs 32098)

Scaffolder	Scaffolds	NG50	Corrected NG50	Corrected E-size	MisAssemblies	Time (hh:mm:ss)
MSR-CA	1476	880130	73094	111918	1068	-
BESST	19760	32921	11796	51309	95	0:11:22
OPERA	-	-	-	-	-	-
SOPRA	-	-	-	-	-	-
SSPACE	13441	37930	29178	51928	146	0:15:38

Table S21: Scaffolders evaluation: MSR-CA

SGA (contigs 930624)

Scaffolder	Scaffolds	NG50	Corrected NG50	Corrected E-size	MisAssemblies	Time (hh:mm:ss)
SGA	30975	74770	58254	89915	19	-
BESST	895510	16477	8476	57214	58	0:53:42
OPERA	928798	2193	2097	3479	39	0:23:18
SOPRA	863294	23696	12464	22246	2253	16:15:22
SSPACE	904940	15488	13618	24789	42	0:38:42

Table S22: Scaffolders evaluation: SGA

SOAPdenovo (contigs 47001)

Scaffolder	Scaffolds	NG50	Corrected NG50	Corrected E-size	MisAssemblies	Time (hh:mm:ss)
SOAPdenovo	38477	368318	56700	99195	268	-
BESST	36920	156638	40858	94068	211	0:07:50
OPERA	-	-	-	-	-	-
SOPRA	-	-	-	-	-	-
SSPACE	35575	86817	37821	75333	205	0:10:46

Table S23: Scaffolders evaluation: SOAPdenovo

Velvet (contigs 133022)

Scaffolder	Scaffolds	NG50	Corrected NG50	Corrected E-size	MisAssemblies	Time (hh:mm:ss)
Velvet	61455	843766	22933	26621	9156	-
BESST	101079	2374	2231	35720	52	0:10:07
OPERA	-	-	-	-	-	-
SOPRA	69050	74772	50130	75378	734	1:27:16
SSPACE	89094	13986	11133	22631	140	0:15:35

Table S24: Scaffolders evaluation: Velvet

2 Proof of Theorem 1

2.1 Function definitions

Since derivation of Theorem 1 is built upon theory from the work of GapEst, we adopt this notation. The following functions can be found, and motivated for, in [3].

- f denotes the standard normal density.
- $p(x \mid c_{min}, c_{max}) = \min\Big\{ \max\{x - 2r + 1, 0\}, c_{min} - r + 1, \max\{|c_{min} + c_{max} - x + 1, 0\}\Big\}$
- $$g(d) = \int_{2r-1}^{c_{min}+c_{max}+1} p(x \mid c_{max}, c_{min}) f(x+d) dx$$
- $$q(d) = f(\tfrac{d+2r-1-\mu_{lib}}{\sigma_{lib}}) + f(\tfrac{c_{max}+c_{min}+d+1-\mu_{lib}}{\sigma_{lib}}) - f(\tfrac{c_{min}+d+r-\mu_{lib}}{\sigma_{lib}}) - f(\tfrac{c_{max}+d+r-\mu_{lib}}{\sigma_{lib}})$$

2.2 Derivation

We have $\sigma_{o|d} = \sqrt{Var(o|d)} = \sqrt{E[o^2|d] - E[o|d]^2}$. We will calculate $E[o^2|d]$ and $E[o|d]$ separately. Note that $o = x - d$ and assume that the gap is known, with length k, *i.e.*, $d = k$. Let h be the gap estimation model introduced in [3] with the corresponding functions p, f, g, I. Also, for overview, the functions are written without their parameters and displaying their dependence of a variable in lowercase, *e.g.*, $p(x-k|c_1, c_2) = p_x$ when integration over variable x, then

$$E[o|d=k] = E[x-k|d=k] = \int_{-\infty}^{+\infty} (x-k) h_x dx$$

$$= \int_{-\infty}^{+\infty} (x-k) \cdot \frac{p_x \cdot f_x}{g_d} dx = \frac{1}{g_d}\Big[\int_{-\infty}^{+\infty} x \cdot p_x f_x dx - k \overbrace{\int_{-\infty}^{+\infty} p_x f_x dx}^{g_d}\Big]$$

$$= \frac{1}{g_d} \overbrace{\int_{-\infty}^{+\infty} x p_x f_x dx}^{:=a} - k = \frac{a}{g_d} - k$$

We let the expression for $E[o|d=k]$ be as it is for now (without deriving a) and go on to calculate $E[o^2|d]$.

$$E[o^2|d=k] = E[(x-k)^2|d=k] = E[x^2-2xk+k^2|d=k]$$

$$= \frac{1}{g_d}\Big[\overbrace{\int_{-\infty}^{+\infty} x^2 p_x f_x dx}^{b} - 2k\overbrace{\int_{-\infty}^{+\infty} x p_x f_x dx}^{a} + k^2 \overbrace{\int_{-\infty}^{+\infty} \cdot p_x f_x dx}^{g_d}\Big]$$

$$= \frac{b}{g_d} - 2k\frac{a}{g_d} + k^2$$

Now we get

$$Var(o|d) = E[o^2|d] - E[o|d]^2 = \Big[\frac{b}{g_d} - 2k\frac{a}{g_d} + k^2\Big] - \Big[\frac{a^2}{g_d^2} - \frac{2ka}{g_d} + k^2\Big] = \frac{b}{g_d} - \frac{a^2}{g_d^2} \tag{1}$$

Now we derive a and b. Note that, using the definition of the normal density f, we have

$$xf_x = -\sigma^2 f'_x + \mu f_x \tag{2}$$

and

$$xf'_x = \mu f'_x - \sigma^2 f''_x - f_x \tag{3}$$

We begin with a.

$$a := \int_{-\infty}^{+\infty} x p_x f_x dx \overset{(2)}{=} -\sigma^2 \int_{-\infty}^{+\infty} p_x f'_x dx + \mu \int_{-\infty}^{+\infty} p_x f_x$$

$$= \sigma^2 \overbrace{\int_{-\infty}^{+\infty} p'_x f_x dx}^{=g'_d} + \mu g_d = \sigma^2 g'_d + \mu g_d$$

where the last equality follows from integration by parts (I.B.P) of the first integral. To see that the first integral equals to $g'(x)$ we refer to the derivation in [3]. We continue with b.

$$
\begin{aligned}
b := \int_{-\infty}^{+\infty} x^2 \cdot p_x f_x dx &\overset{(2)}{=} \int_{-\infty}^{+\infty} x\big(-\sigma^2 f'_x + \mu f_x\big) p_x dx \\
&= -\sigma^2 \int_{-\infty}^{+\infty} x f'_x p_x dx + \mu \int_{-\infty}^{+\infty} x f_x p_x dx \overset{(3),(2)}{=} \\
-\sigma^2 \int_{-\infty}^{+\infty} (\mu f'_x - \sigma^2 f''_x - f_x) p_x dx &+ \mu \int_{-\infty}^{+\infty} (-\sigma^2 f'_x + \mu f_x) p_x dx \\
= -2\sigma^2 \mu \overbrace{\int_{-\infty}^{+\infty} f'_x p_x dx}^{I.B.P \Rightarrow -g'_d} + \sigma^4 \int_{-\infty}^{+\infty} f''_x p_x dx &+ (\mu^2 + \sigma^2) \overbrace{\int_{-\infty}^{+\infty} f_x p_x dx}^{g_d} \\
&= 2\sigma^2 \mu g'_d + (\mu^2 + \sigma^2) g_d + \sigma^4 r_d
\end{aligned}
$$

Here,

$$
\begin{aligned}
r(d) &= \sigma^4 \int_{-\infty}^{+\infty} f''_x p_x dx \\
= \phi\Big(\frac{d + 2r - 1 - \mu_{lib}}{\sigma_{lib}}\Big) &+ \phi\Big(\frac{c_{max} + c_{min} + d + 1 - \mu_{lib}}{\sigma_{lib}}\Big) \\
-\phi\Big(\frac{c_{min} + d + r - \mu_{lib}}{\sigma_{lib}}\Big) &- \phi\Big(\frac{c_{max} + d + r - \mu_{lib}}{\sigma_{lib}}\Big)
\end{aligned}
$$

second equality being obtained by integration by parts two times with antiderivative of f and derivative of p. Now, from Equation (1) we get

$$
\begin{aligned}
Var(o|d) &= \frac{2\sigma^2 \mu g'_d + (\mu^2 + \sigma^2) g_d + \sigma^4 r_d}{g_d} - \frac{(\sigma^2 g'_d + \mu g_d)^2}{g_d^2} \\
&= \sigma^2 + \frac{r(d)\sigma^4}{g(d)} - \frac{g'(d)^2 \sigma^4}{g(d)^2}
\end{aligned}
$$

Which is equivalent to the result we want to prove since a variance is always positive, *i.e.*, the square root operation is well defined.

References

[1] Tatiana Belova, Bujie Zhan, Jonathan Wright, Mario Caccamo, Torben Asp, Hana Šimková, Matthew Kent, Christian Bendixen, Panitz, Frank, Sigbjørn Lien, Jaroslav Doležel, Odd-Arne Olsen, and Simen R Sandve. Integration of mate pair sequences to improve shotgun assemblies of flow-sorted chromosome arms of hexaploid wheat. *BMC Bioinformatics*, 14(222), 2013.

[2] D. Earl, K. Bradnam, J. St John, A. Darling, D. Lin, J. Fass, H.O. Yu, V. Buffalo, D.R. Zerbino, M. Diekhans, N. Nguyen, P.N. Ariyaratne, W.K.

Sung, Z. Ning, M. Haimel, J.T. Simpson, N.A. Fonseca, I. Birol, T.R. Docking, I.Y. Ho, D.S. Rokhsar, R. Chikhi, D. Lavenier, G. Chapuis, D. Naquin, N. Maillet, M.C. Schatz, D.R. Kelley, A.M. Phillippy, S. Koren, S.P. Yang, W. Wu, W.C. Chou, A. Srivastava, T.I. Shaw, J.G. Ruby, P. Skewes-Cox, M. Betegon, M.T. Dimon, V. Solovyev, I. Seledtsov, P. Kosarev, D. Vorobyev, R. Ramirez-Gonzalez, R. Leggett, D. MacLean, F. Xia, R. Luo, Z. Li, Y. Xie, B. Liu, S. Gnerre, I. MacCallum, D. Przybylski, F.J. Ribeiro, S. Yin, T. Sharpe, G. Hall, P.J. Kersey, R. Durbin, S.D. Jackman, J.A. Chapman, X. Huang, J.L. DeRisi, M. Caccamo, Y. Li, D.B. Jaffe, R.E. Green, D. Haussler, I. Korf, and B. Paten. Assemblathon 1: a competitive assessment of de novo short read assembly methods. *Genome Research*, 21(12):2224–2241, Dec 2011.

[3] Kristoffer Sahlin, Nathaniel Street, Joakim Lundeberg, and Lars Arvestad. Improved gap size estimation for scaffolding algorithms. *Bioinformatics*, 17:2215–2222, 2012.

[4] S. L. Salzberg, a. M. Phillippy, a. V. Zimin, D. Puiu, T. Magoc, S. Koren, T. Treangen, M. C. Schatz, a. L. Delcher, M. Roberts, G. Marcais, M. Pop, and J. a. Yorke. GAGE: A critical evaluation of genome assemblies and assembly algorithms. *Genome Research*, 22(3):557–567, December 2012.

[5] Francesco Vezzi, Giuseppe Narzisi, and Bud Mishra. Feature-by-feature – evaluating de novo sequence assembly. *PLoS ONE*, 7(2):e31002, February 2012.

[6] Francesco Vezzi, Giuseppe Narzisi, and Bud Mishra. Reevaluating assembly evaluations with feature response curves: Gage and Assemblathons. *PLoS ONE*, 7(12):e52210, 2012.
